# Cereal Crop Proteomics: Systemic Analysis of Crop Drought Stress Responses Towards Marker-Assisted Selection Breeding

**DOI:** 10.3389/fpls.2017.00757

**Published:** 2017-06-02

**Authors:** Arindam Ghatak, Palak Chaturvedi, Wolfram Weckwerth

**Affiliations:** ^1^Department of Ecogenomics and Systems Biology, University of ViennaVienna, Austria; ^2^Vienna Metabolomics Center, University of ViennaVienna, Austria

**Keywords:** wheat, rice, maize, barley, sorghum, pearl millet, proteomics, climate change

## Abstract

Sustainable crop production is the major challenge in the current global climate change scenario. Drought stress is one of the most critical abiotic factors which negatively impact crop productivity. In recent years, knowledge about molecular regulation has been generated to understand drought stress responses. For example, information obtained by transcriptome analysis has enhanced our knowledge and facilitated the identification of candidate genes which can be utilized for plant breeding. On the other hand, it becomes more and more evident that the translational and post-translational machinery plays a major role in stress adaptation, especially for immediate molecular processes during stress adaptation. Therefore, it is essential to measure protein levels and post-translational protein modifications to reveal information about stress inducible signal perception and transduction, translational activity and induced protein levels. This information cannot be revealed by genomic or transcriptomic analysis. Eventually, these processes will provide more direct insight into stress perception then genetic markers and might build a complementary basis for future marker-assisted selection of drought resistance. In this review, we survey the role of proteomic studies to illustrate their applications in crop stress adaptation analysis with respect to productivity. Cereal crops such as wheat, rice, maize, barley, sorghum and pearl millet are discussed in detail. We provide a comprehensive and comparative overview of all detected protein changes involved in drought stress in these crops and have summarized existing knowledge into a proposed scheme of drought response. Based on a recent proteome study of pearl millet under drought stress we compare our findings with wheat proteomes and another recent study which defined genetic marker in pearl millet.

## Introduction

**Figure 1:**
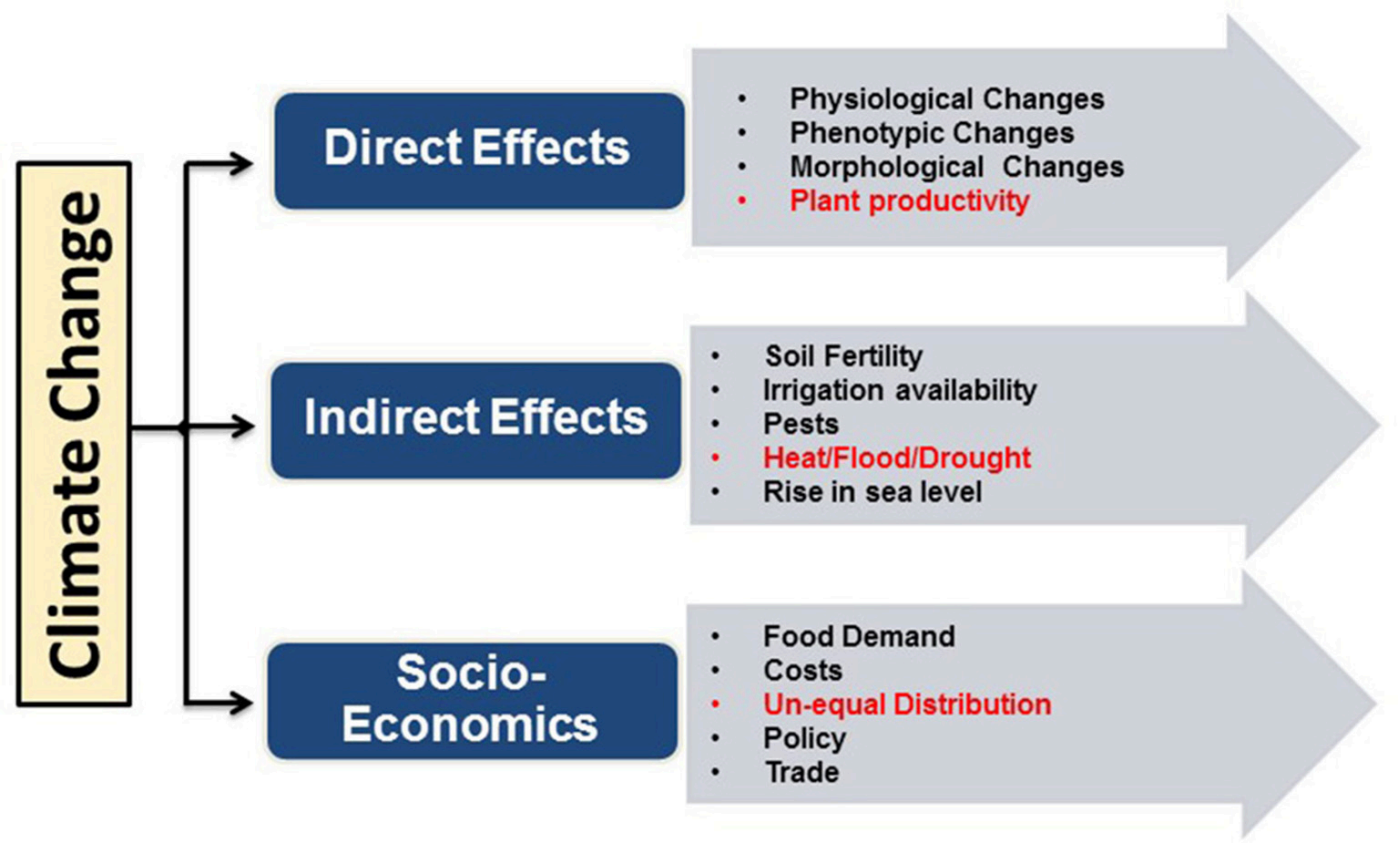
**Impact of climate change on agriculture**.

An alarming situation across the globe at present is the rise in global warming, which has a direct impact on climatic changes like decrease in land ice (287 billion metric tons/year), rise in carbon dioxide (401.58 ppm), temperature rise (1.4°F since 1880), drought, depletion in fresh water level (35% per decade), melting of ice (13.3% per decade), rise in the sea level (3.24 mm/year), forest fires and most important food shortage caused by yield reduction (http://climate.nasa.gov/evidence/). Based on the IPCC report, the average global surface temperature will increase in the range of 1.1–6.4°C by the end of this century. The certainty that climate will continue to change in the future thus raises many questions related to food security. Currently, researchers and farmers seek to sustain the impressive historical gains by improved genetics and agronomic management of major food crops (Lobell and Gourdji, 2012). An overview of the effects of climate change on agricultural productivity is illustrated in Figure 1.

Abiotic stresses like drought and high temperature severely hamper crop productivity and sustainability to a great extent. Drought is one of the most critical threat to agriculture productivity worldwide as we face the scarcity of water resources throughout the globe. The severity of drought is unpredictable as it relies on several factors like occurrences and distribution of rainfall, evaporation rate and moisture retaining capacity of the soil (Wery et al., 1994). Soil water deficit condition reduces crop yield by the following three main mechanisms: (1) reduction in canopy absorption of photosynthetically active radiation (PAR) (2) decreased radiation use efficiency (RUE) (3) decreased harvest index (HI) (Earl and Davis, 2003).

Application of “Omics” technologies like genomics, transcriptomics, proteomics and metabolomics in the field of agriculture will provide consistency and predictability in plant breeding processes, producing high quality food crops that are resistant to biotic/abiotic stress and render high nutritive value (Tester and Langridge, 2010; Roy et al., 2011; Weckwerth, 2011a,b; Beddington et al., 2012; Parry and Hawkesford, 2012; Boggess et al., 2013). Omics analysis is part of a systems biology approach in order to understand complex interactions between genes, proteins and metabolites within the resulting phenotype (Weckwerth, 2003, 2011a,b; Weckwerth et al., 2004). Consequently, this integrative understanding relies on genome analysis, bioanalytical approaches as well as bioinformatics (Roy et al., 2011; Weckwerth, 2011a).

Genomic applications provide a systematic knowledge based approach for crop plant biology and hence enable precise and control methods for molecular and marker assisted breeding accelerating the process of development of new and resistant crop varieties (Ahmad et al., 2012). The first plant genome to be sequenced (in 2000) was *Arabidopsis thaliana*, a small annual herb of the Brassicaceae family with 25,498 genes (Kaul et al., 2000). Following this, many other plant genomes have been added to the list which includes barley, melon, orange, tomato, potato, cacao, watermelon, and many more. The availability of these comprehensive public sequence databases has a strong impact on proteome research, which in turn significantly helps to develop potential biomarkers (Weckwerth, 2011a).

**Figure 2:**
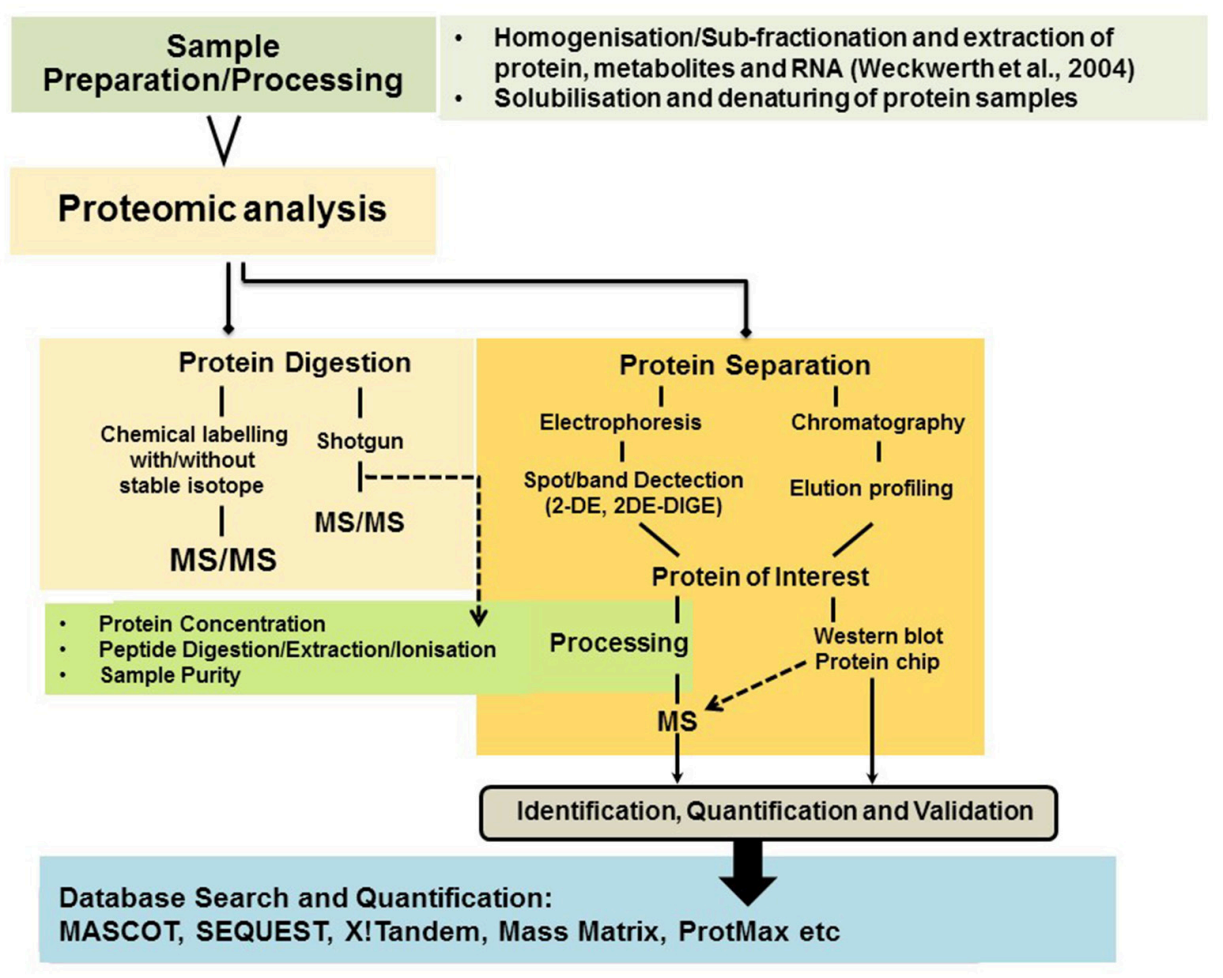
**Overview of potential proteomics strategies**.

Proteins are the central biomolecules that are responsible for all cellular functions in the living organism. Proteomics can be defined as efficient and systematic high-throughput identification of proteins present in tissues, cells or the sub-cellular compartment. This technique allows qualitative and quantitative measurements of large number of proteins which are directly involved/influence cellular biochemistry. Proteomic approaches provide information about protein concentrations, post translational modification (PTMs), protein-protein interaction, regulatory functions of proteins coded by genes and structure associated with stress tolerance. A schematic representation of proteomics strategies is provided in Figure 2.

In order to study dynamic changes in the proteome pattern, two-dimensional gel electrophoresis (2-DE) is a classical method routinely applied. It is based on two independent parameters: the isoelectric point (pI) and molecular mass. In order to overcome limitations such as gel-to-gel variation and reproducibility, a technique known as difference in gel electrophoresis (DIGE) has been developed (Görg et al., 2004; Wittmann-Liebold et al., 2006). In this technique each protein sample is labeled at lysine residues with different fluorophores. The samples are then mixed and separated on the same gel in order to increase identification sensitivity of differentially expressed proteins. Gel based proteomics techniques, novel mass spectrometers (MS) and strategies like top-down or bottom-up have been developed in recent years which comprehensively allow us to characterize proteomes of an organisms. Advances in mass spectrometers (MS) over the years have established MS as a primary tool for protein identification (Glinski and Weckwerth, 2006). It consists of mass analyzer, ion sources and a detector which measures mass-to-charge (m/z) ratios (Han et al., 2008). Coupling of soft ionization techniques to mass spectrometers (MS) have also revolutionized proteome analysis, for example matrix assisted laser desorption/ionization (MALDI) and electrospray ionization (ESI) (Karas and Hillenkamp, 1988; Fenn et al., 1989).

Gel free protein separation and second generation proteomic techniques like shotgun proteomics [multidimensional protein identification technology (MudPIT)], quantitative proteomic approaches such as isotope-code affinity tags (ICATs), targeted mass tags (TMTs), isobaric tags for relative and absolute quantitation (iTRAQ) and stable-isotope labeling of amino acids in cell culture have been widely used for comparative proteomic studies (Oda et al., 1999; Matros et al., 2011). Due to the development of robust and reproducible hyphenated techniques such as liquid-chromatography coupled to mass spectrometry label-free-shotgun proteomics has developed into a cornerstone of quantitative proteomics and phosphoproteomics studies which cannot be reviewed here in detail. However, many recent studies from our lab have provided lots of insights in proteome dynamics and adaptation strategies of various plant species and families in stress physiology, development and even field studies of crop plants (Weckwerth et al., 2004; Wienkoop et al., 2004, 2006, 2008; Morgenthal et al., 2005; Hoehenwarter et al., 2008, 2011, 2013; Weckwerth, 2008; Chen et al., 2010; Cerný et al., 2013; Chaturvedi et al., 2013, 2015, 2016; Valledor et al., 2013, 2014; Ischebeck et al., 2014; Paul et al., 2015; Ghatak et al., 2016, in press; Liu et al., 2016; Nukarinen et al., 2016; Wang et al., 2016a,b). Especially, the analysis of posttranslational modifications of proteins is an exclusive approach in proteomics and cannot be covered by any other method. Quantitative phosphoproteomics allows insights into *in vivo* signaling perception and transduction and reveals processes of translational activity which are otherwise not recognizable (Nukarinen et al., 2016). Recently, we have further demonstrated in a kinetic modeling approach that without any transcriptional activity sugar homeostasis can be rapidly adjusted by 10 orders of magnitude (Nägele and Weckwerth, 2014). Thus, the analysis of proteins and their activities—as well as the corresponding metabolites—is of utmost importance to understand any metabolic adjustment or regulation and cannot be predicted from genome information or by genomic/genetic tools (Weckwerth, 2011b).

In this review, we focus on proteomics studies of cereal crop plants (i.e., wheat, rice, maize, barley, sorghum, and pearl millet) under drought stress which provide a broad spectrum of involved drought responsive protein (DRP) markers in cereal crops.

## Drought stress

Plants are sessile organisms which response to drought stress via complex biochemical, physiological, morphological, anatomical as well as short and long-term developmental and growth-related adaptation processes. The common drought responsive mechanism comprises several characteristics: (1) Drought escape via completing plant life cycle before severe water stress conditions (e.g., early flowering). (2) Drought avoidance via enhancing water taking capacity (e.g., developing root systems or conserving water by reducing transpiration such as closure/reduction of stomata and leaf area). (3) Drought tolerance via improving osmotic adjustment and increasing cell wall elasticity to maintain tissue turgidity. (4) Drought resistances via altering metabolic pathways under severe water stress condition. (5) Drought abandon by reducing / removing a part (e.g., shedding mature leaf under stress condition). (6) Drought prone biochemical-physiological traits for resistant plant under long term drought stress (e.g., genetic mutation and modification). All these processes maybe involved simultaneously in plant responses to drought stress and followed by re-watering (Mitra, 2001). This complexity cannot be resolved without comprehensive data mining strategies involving genome-scale metabolic reconstruction and modeling as well as statistical multivariate methods developed in the framework of systems biology (Weckwerth, 2003, 2008, 2011a,b; Nukarinen et al., 2016)

**Figure 3:**
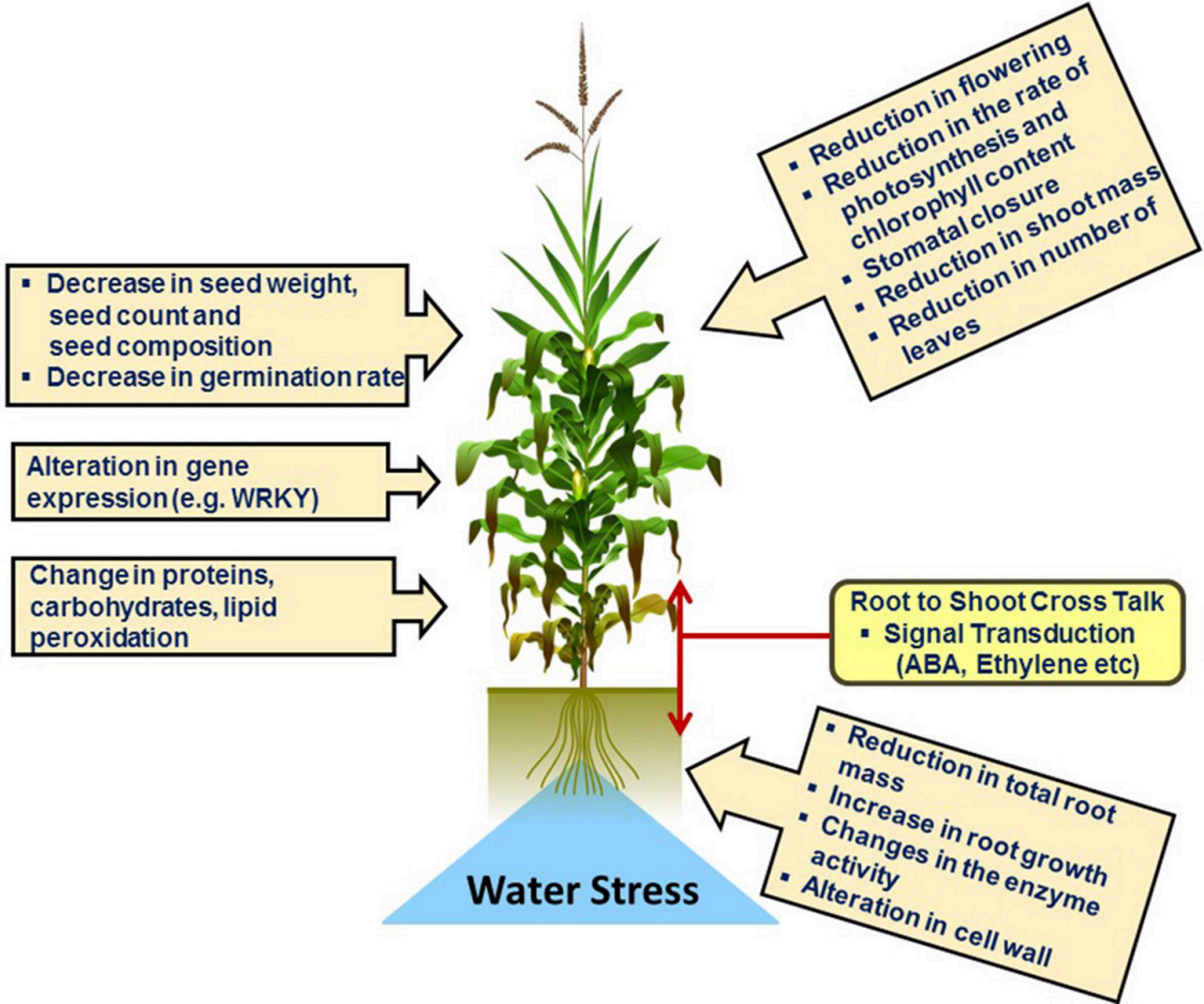
**Physiological and Morphological changes in plants under water deficit condition**.

**Figure 4:**
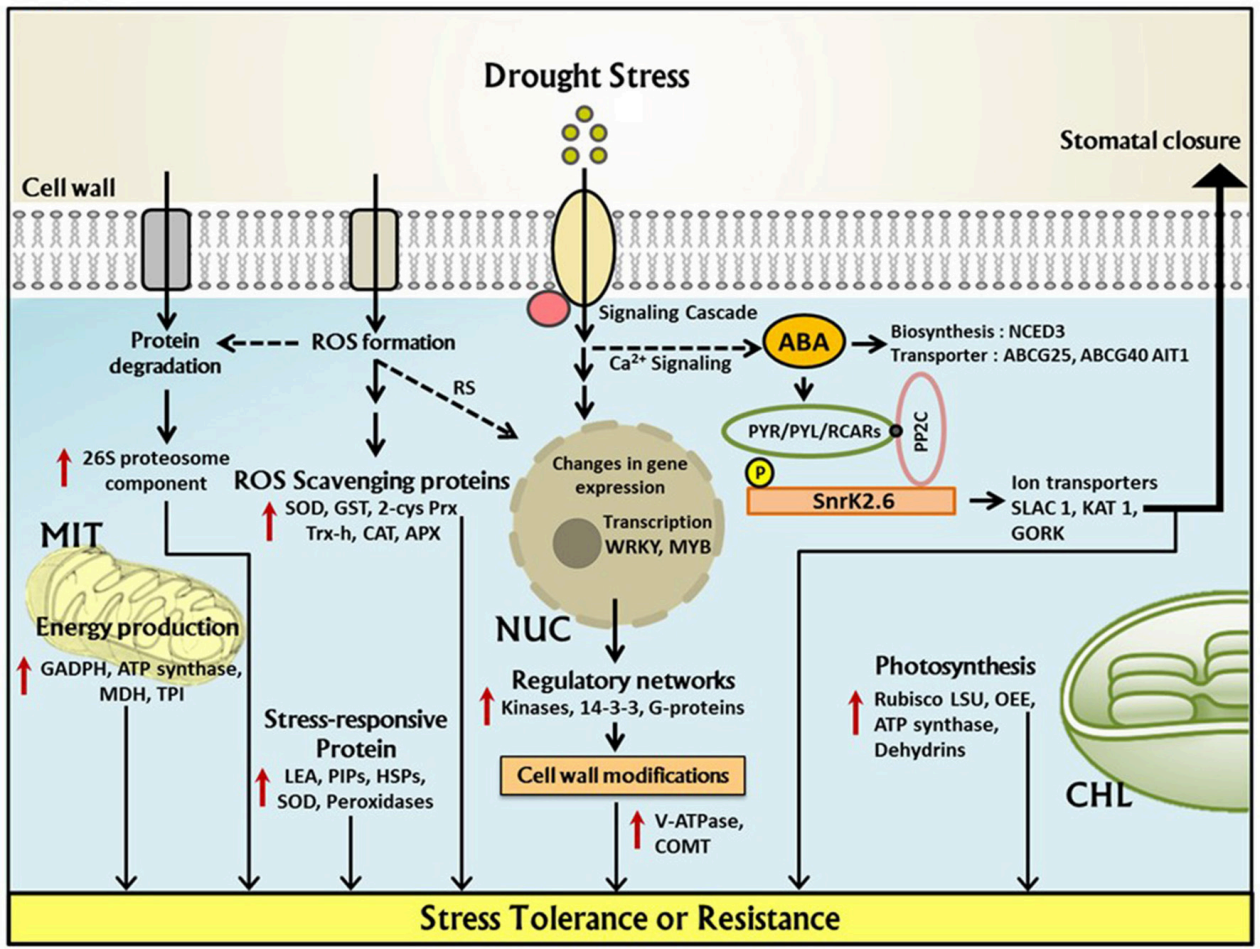
**Underlying regulatory network of drought stress response in cereal crop proteomes**. Within the plant cell, upregulation of key responsive proteins under drought stress has been demonstrated which are important for plant stress tolerance or resistance. NUC, Nucleus; MIT, Mitochondria; CHL, Chloroplast; 2Cys-Prx, 2-cysteineperoxiredoxin; APX, ascorbate peroxidase; SOD, superoxide dismutase; GST, glutathione S-transferase; Trx-h, thioredoxin h; COMT, caffeoyl-coenzyme A O-methyl transferase; LEA, Late embryogenesis-abundant (protein); TPI, triose phosphate isomerase; V-ATPase, vacuolar ATPase; RubisCO LSU, RubisCO large subunit; GAPDH, glyceraldehyde-3-phosphatedehydrogenase; ABA, abscisic acid; SnRK, sucrose non-fermenting-related protein kinase; MDH, malate dehydrogenase; PP2C, Protein phosphatase 2C; OEE, oxygen evolving enhancer(protein); HSPs, heat shock proteins; ROS, Reactive oxygen species; CAT, catalase; NCED, 9-cis-epoxycarotenoid dioxygenase; SLAC 1, Slow anion channel-associated 1; GORK, Gated outwardly-rectifying K+ channel; KAT 1, Potassium channel; RS, ROS induced signaling; PYR/PYL/RCAR, PYRABACTIN RESISTANCE1 (PYR1)/PYR1-LIKE (PYL)/REGULATORY COMPONENTS OF ABA RECEPTORS.

Under drought stress the first fundamental response of plants is the closure of stomata in order to prevent transpiration which in turn decreases water loss. Physiological and morphological changes in plants under drought stress conditions are illustrated schematically in Figure 3. Closure of stomata decreases the inflow of CO_2_ into the leaves which leads to the formation of reactive oxygen species (ROS). Several experiments have proven that stomatal response is more often linked to soil moisture content which suggests that stomatal behavior largely depends upon chemical signals in root-shoot communication, e.g., abscisic acid (ABA) (Osakabe et al., 2014). Endogenous ABA is rapidly produced under drought stress which triggers many physiological responses and regulates signal transduction networks. A recent study revealed that in *Arabidopsis* expression of 9-cis-epoxycarotenoid dioxygenase 3 (*NCED3*) is rapidly induced under drought stress in vascular tissue specific manner. *NCED3* catalyzes a key step in ABA biosynthesis (Iuchi et al., 2001; Endo et al., 2008). In Figure 4 these key regulatory signal perception and transduction processes as well as important protein marker for drought stress adaptation revealed by the reviewed proteomics studies below are summarized.

Accumulated ABA under drought stress in the vascular tissue is transported to guard cells via passive diffusion and by a specific transporter. Three independent ABA transporter were isolated from *Arabidopsis* which includes ABCG25, ABCG40 (member of ABC transporter family) and AIT1/NRT1.2/ NPF4.6 (nitrate transporter family). Under drought stress transcription of ABCG25 is induced by ABA which exhibit vascular tissue specificity whereas ABCG40 is expressed in guard cells providing evidence that ABA is synthesized in vascular tissues and imported into guard cell via these transporters (Kuromori et al., 2010; Kanno et al., 2012). The ABA signals are first recognized by several receptors which include (PYRABACTIN RESISTANCE/ PYRABACTIN RESISTANCE–LIKE/REGULATORY COMPONENT OF ABA RESPONSE) like proteins (Klingler et al., 2010). This protein belongs to the START-domain superfamily and has soluble ligand-binding properties. ABA binding to PYR/PYL/RCAR leads to inactivation of type 2C protein phosphatases (PP2Cs) such as ABSCISIC ACID INSENSITIVE 1 (ABI1) and its close homolog ABI2 (Nishimura et al., 2010). Other receptor proteins includes GCR2 (G protein coupled receptor; Mustilli et al., 2002) and GPCR (GPROTEIN COUPLED RECEPTOR)-TYPE G PROTEINS GTG1 and GTG2 (Pandey et al., 2009; Figure 4).

Various genes/proteins are expressed and translated in response to water deficit conditions. Most of the stress proteins are water soluble, hence contribute to stress tolerance phenomena by hydrating various cellular structures (Osakabe et al., 2014). Several studies have identified conserved and species-specific drought responsive genes/proteins which include membrane stabilizing proteins, late embryogenic abundant proteins (LEA) which increases water binding capacity and also referred as dehydrins. Several heat shock proteins were also identified which play a major role in stabilizing protein structure. Low molecular weight heat shock proteins are translated in response to environmental stress particularly during high temperature stress. These proteins also act as molecular chaperones which participate in ATP dependent protein unfolding reaction and prevent denaturation during stress condition. Additionally, several transcription factors were also identified which include MYB, MYC, DREB/CBF, ABF/AREB, NAC, WRKY etc. Further SnRK2 also regulates and provide adaptive response under drought stress (Stockinger et al., 1997; Sakuma et al., 2006; Nakashima et al., 2009b; Tran et al., 2010; Umezawa et al., 2013, 2014; Saruhashi et al., 2015). SnRK2 deficit mutants do not exhibit ABA-mediated stomatal closure activity and display wilty phenotype under dehydration stress conditions (Fujii and Zhu, 2009; Fujita et al., 2009). Other SnRK2 like SnRK2.6/OST1 physically interacts with ABI1 and ABI2 under stress condition; it acts as a positive regulator in ABA-induced stomatal closure (Nakashima et al., 2009a). A study reported by Hu and co-workers demonstrated expression of SNAC1 (Stress responsive NAC1) in rice guard cells, over expression of this gene increases ABA sensitivity and stomatal closure (Hu et al., 2006).

## Cereal crop proteomics under drought stress

Recent statistics estimates that approximately 925 million people suffer from starvation on this globe (Karimizadeh et al., 2011) and it is expected that nearly two billion people will be added by the year 2050 (UN, 2012). In order to eradicate or avoid this situation we need to increase the food production and supply significantly. This can be achieved by integrating several elements which include plant breeding tools for crop improvement (Roy et al., 2011; Beddington et al., 2012).

One of the major shortcomings faced by the plant breeders for crop improvement programs is the limited gene pool of domesticated crops. Identification of potential genes across the plant kingdom can play a major role in the improvement of crop traits. This information can be extensively obtained from advancement in the field of molecular biology which includes genomics, proteomics and PTM-proteomics as discussed above. The identified new genes can be placed by smart breeding into a desired crop to enhance the productivity of the crop and help in the current scenario.

The following sections will focus on different food crops like wheat, rice, maize, barley, sorghum and pearl millet providing insights of the drought stress proteomes and identification of putative biomarker aiming for the understanding of systemic drought responses and subsequent selection of markers for the process described above. Table 1 provides details of the studies on ceral crop drought stress proteomics providing a list of proteins which have shown significant changes.

**Table 1:** **List of proteomic studies focused on drought stress in cereal crops**.

**Cultivar**	**Plant material**	**Method**	**Protein identification (TI: total identification, DRP: Drought responsive proteins)**	**Up-regulated protein candidates**	**References**
**WHEAT**					
*Triticum aestivum* L. Arvand, Khazar-1, and Kelk Afghani	Seeds	2-DE, MALDI-TOF/TOF	TI: 121 DRP: 57	Trx h, 1-Cys peroxiredoxin, GST, PDI, LEA, sHSP17, HSP70	Hajheidari et al., 2007
Durum wheat cv. Ofanto	Leaves	2-DE-PAGE, MALDI-TOF-MS	DRP: 36	Carbonic anhydrase, RubisCO LSU	Caruso et al., 2009
*Triticum aestivum* L. cv. China-108, cv. Yennon-78, cv. Norin-61,cv. Kantou-107	Grains	MALDI-TOF-MS	DRP: 33	ABA responsive proteins, LEA, cys-peroxiredoxin, elf proteins, cyclin-dependent kinase like, MYB, lipid transfer proteins and WRKY	Kamal et al., 2010
*Triticum aestivum* L. cv. Shanrong No. 3, cv. Jinan 177	Roots and leaves	MALDI-TOF/TOF-MS	DRP: 93 Roots, DRP: 65 leaves	G protein -subunit-like protein, Rubisco LSU, Serine/threonine kinase, GTP-binding protein, Glutathione transferase F4	Peng et al., 2009
Australian wheats cv. Kukri, cv. excalibur, cv. RAC875	Leaves	nanoLC-MS/MS	TI: 1299	CAT, Cu/Zn-SOD, Mn-SOD	Ford et al., 2011
*Triticum aestivum* L. cv. Nesser, cv. Opata M85	Roots	nanoLC-MS/MS, iTRAQ	TI: 1656, ABA- Responsive Proteins: 805	LEA, PP2C, HSP70, HSP90, 14-3-3, G-proteins; V-ATPase; β- expansin, porins	Alvarez et al., 2014
*Triticum aestivum* L. SERI M 82, SW89.5193/kAu2	Roots and leaves	2DE, nano-LC-MS/MS	TI: 237 DRP: 49	16.9 kDa HSP,GST, Germin-like protein, 14-3-3 protein, ATP synthase	Faghani et al., 2015
*Triticum boeoticum*	Roots and leaves	2DE, MALDI-TOF/TOF	Roots DE: 98, leaves DRP: 85	UDP-glucose/GDP-mannose dehydrogenase, transketolase, transaldolase-like protein, ribulose-phosphate 3-epimerase, ATP synthase beta subunit, thioredoxin-disulfide reductase	Liu et al., 2015
*Triticum aestivum* L. cv. CS, cv. HX-10	Roots, Leaves, ISRL	2 DE, MALDI-TOF/TOF	DRP: Roots 44, leaves 78 and ISRL 34	14-3-3, calreticulin-like protein, HSP90, 70, 23.5, RuBisCO LSU.	Hao et al., 2015
*Triticum aestivum* L. cv. HX10 and NC 47	Leaves	LTQ-Orbitrap XL MS	TI: HX10 - 173, NC 47-227 DRP: 31	WCOR615, monosaccharide sensitive protein 2 (MSSP2), wheat aluminum induced 7 (WALI7), Serine/threonine-protein kinase PRP4-like protein, TaABI5, PP2C, H+ -ATPase	Zhang et al., 2014
*Triticum aestivum* L. cv. Xihan No. 2, cv. Longchun 23	Leaves	2-DE, MALDI-TOF/TOF MS	TI: 387 DRP: 148	Acid phosphatase, glyceraldehyde-3-phosphate dehydrogenase, peptidyl-prolyl cis-trans isomerase, proteasome subunit alpha, voltage dependent anion channel and S-like RNase	Cheng et al., 2016
Durum wheat cv. Kiziltan, *T. dicoccoides* lines TR39477, TTD22	Leaves	2DE, nanoLC-ESI-MS/MS	TI: 75 DRP: 11	TPI, ATP synthase CF1, β-1,3-glucanase, β-1,4-glucanase, XET	Budak et al., 2013
*Triticum aestivum* L. cv. Yumai 34	Leaves	2DE, MALDI-TOF-TOF MS	DRP: 76	14−3−3, Ferredoxin-NADP(H) oxidoreductase, SET-domain transcriptional regulator, protein agamous-like 26, glutathione S-transferase 1	Kang et al., 2012
*Triticum aestivum* L. cv. Jimai 22	Grains	2DE, MALDI-TOF-TOF MS	TI: 136 DRP: 89	Cu/Zn SOD, glutathione transferases, myo-inositol-1-phosphate synthase, ATP synthase, HSP70	Qin et al., 2014
*Triticum durum* Desf. cv. Waha, Oued Zenati, cv. Djenah Khetifa	Callus	D-2DE, MALDI-TOF-TOF MS	DRP: 16	Glyceraldehyde-3-phosphate dehydrogenase, Globulin 1S, 3A, peroxidases	Kacem et al., 2016
*Triticum turgidum* spp. Durum cv. Ciccio, cv. Svevo	Grains	2 DE, nanoLC-ESI-IT-MS/MS	DRP: 24	HMW glutenin x-type subunit Bx7, ω-5-gliadin, triticin	Giuliani et al., 2015
*Triticum aestivum* L. cv. Kauz, cv. Janz	Grains	2 DE, MALDI-TOF-TOF MS	DRP: 153	Sucrose synthase, triticin, catalase isozyme 1, WD40 repeat protein, LEA and alpha-amylase inhibitors, GAPDH	Jiang et al., 2012
*Triticum aestivum* L. cv. Chinese Spring, Ningchun 4	Grains	2 DE, MALDI-TOF-TOF MS	TI: 152 Unique proteins 58	Sucrose synthase, triticin precursor, cytosolic malate dehydrogenase, cytoplasmic aldolase, GAPDH, cytosolic 3-phosphoglycerate kinase	Ge et al., 2012
*Triticum aestivum* L. cv. Vinjett	Leaves	2DE, MALDI-TOF MS, MS/MS	TI: 400	Rubisco LSU, peptidyl-prolyl cis-trans isomerase CYP38, 4-nitrophenyl phosphatase, PSII stability/assembly factor HCF136, Thioredoxin-like protein, CDSP32	Wang et al., 2014
*Triticum aestivum* L. cv. Vinjet	Grains	2DE, MALDI-TOF-TOF MS	TI:250, 100	LEA, peroxiredoxins and a-amylase/trypsin inhibitors, 3-phosphoglycerate kinase	Yang et al., 2011
*Triticum turgidum* L. var. durum cv. Borgia, MD-597	Leaves	2DE, MALDI-TOF-TOF MS	TI:182	Oxygen-evolving enhancer protein 2, catalase-1 (CATA1)	Peremarti et al., 2014
*Triticum aestivum* L. cv. N14, cv. N49	Stem	2DE, MALDI TOF/TOF or nano LC-ESI-Q-TOF MS/MS	DRP: 135	Rubisco LSU, MFP1 attachment factor 1, Tubulin folding cofactor A, thioredoxin H-type (TRX-H), Glyceraldehyde 3-phosphate dehydrogenase	Bazargani et al., 2011
*Triticumaestivum* L. cv. Chinese Spring, cv. NC47	Leaves	2DE, MALDI-TOF/TOF-MS	DRP: 101 (Unique proteins: 77)	Dehydroascorbate reductase (DHAR), ascorbate peroxidase (APX), Cu/Zn superoxide dismutase (SOD), 2-Cys peroxiredoxin and a fibrillin-like protein, chitinase 2, HSP70, RuBisCO LSU	Cheng et al., 2015
**RICE**					
*Oryza sativa* L. cv. IR64	Roots	nanoLC-MS/MS	TI: 1487	sHSP II 17.8, Root PR-10, Tim13, CAT-A, Peroxidase 22	Mirzaei et al., 2012b
*Oryza sativa* L. cv. Nipponbare	Leaves	nanoLC-MS/MS	TI: 1548	PIPs protein, GTP binding protein, Ras-related protein RIC1, ethylene-responsive small GTP, L-ascorbate peroxidase 2	Mirzaei et al., 2012a
*Oryza sativa* L. var. Rasi	Apoplastic	2-DE, LC-MS/TOF	TI: 250 DRP: 192	Thioredoxin M, 2-cys peroxiredoxin, Hsp70/DnaK, 14-3-3 protein, oryzacystatin, phosphoribulokinase	Pandey et al., 2010
*Oryza sativa* L. cv. CT9993, cv. IR62266	Leaves	2-DE, MALDI-MS/ESI-Q-TOF-MS/MS	DRP: 42	actin depolymerizing factor, translational initiation factor EF-Tu, rubisco activase isoforms, chloro-plast fructose-1,6-bisphosphate aldolase, glutathione dehydro ascorbate reductase	Salekdeh et al., 2002
*Oryza sativa* L. cv. Nipponbare	Leaves	2-DE	DRP: 18, Phosphoproteins: 10	ABA, LEA, chloroplast Cu–Zn SOD, Ribosomal protein, NAD-malate dehydrogenase	Ke et al., 2009
*Oryza sativa* L. IR64, Moroberekan	Anthers	2-DE, MALDI-TOF-MS/ESI-TOF MS/MS	TI: 93 DRP: 35	Peroxiredoxin, Ascorbate peroxidase, Putative actin binding protein, Glutathione S-transferase 2, Adenosine kinase 2	Liu and Bennett, 2011
*Oryza sativa* L.cv. Heena	Roots	2-DE, MALDI-TOF MS-MS	TI: 125 DRP: 78	malate dehydrogenase, succinyl-CoA, pyruvate dehydrogenase, cysteine synthase, prolyl endopeptidase	Agrawal et al., 2016
*Oryza sativa* L. cv. Nipponbare, IAC1131	Leaves	nanoLC–MS/MS, LTQ-XL ion-trap MS	DRP: 40 (Label free) DRP: 114 (TMT label)	ClpB1, Hsp17.9, Hsp18.6, HSP 70, RuBiscCO LSU, chaperone protein ClpD1	Wu et al., 2016
*Oryza sativa* L. ssp. Indica cv. Zhenshan97B, ssp. Japonica cv. IRAT109	Leaves	2-DE, MALDI-TOF MS	DRP: 17 (Zhenshan) DRP: 20 (IRAT109)	Putative glycine hydroxymethyltransferase, Ribulose bisphosphate carboxylase/oxygenase activase, chloroplast Cu–Zn SOD, Dehydroascorbate reductase, Putative ATP synthase beta subunit	Ji et al., 2012
*Oryza sativa* L. ssp. Japonica cv. Yongyou 8	Spikelets	iTRAQ, LC-MS/MS	TI: 1207 DRP: 185	Histone H3, Ribulose bisphosphate carboxylase, Thioredoxin H-type, Ras-related protein RIC1	Dong et al., 2014
*Oryza sativa* L. var. japonica	Leaves	2-DE, MALDI-TOF/TOF MS	DRP: 15	Ribulose-1,5-bisphosphate carboxylase/oxygenase activase, Drought-induced S-like ribonuclease, Putative remorin 1 Protein, Ascorbate peroxidase	Rabello et al., 2014
*Oryza sativa* L. cv. IRAT109	Leaves	2-DE, MALDI-TOF/TOF MS	DRP: 71	HSP 90, HSP70, Putative chaperonin 60 β, Dehydroascorbate reductase, Putative peptide methionine sulfoxide reductase	Shu et al., 2011
*Oryza sativa* L. cv. Zhonghua 8, Nipponbare	Seedling	2-DE, MALDI-TOF MS	DRP: 15	dnaK-type molecular chaperone, Endosperm luminal binding protein (BiP), Uroporphyrinogen decarboxylase	Zang and Komatsu, 2007
*Oryza sativa* L. cv. IR64	Leaves	nanoLC−MS/MS LTQ-XL	TI: 1383	Cyt b6-f complex Fe-S subunit, Ferritin, Succinate dehydrogenase, Putative hexose transporter, proteasome subunit beta type, drought induced S-like ribonuclease, HSP 101, succinate dehydrogenase, ATP-dependent Clp protease	Mirzaei et al., 2014
*Oryza sativa* L. cv. IR64	Peduncles	2-DE, MALDI-TOF MS	DRP: 31	Putative actin-binding protein, LEA type-1 protein, GSH-dependent dehydroascorbate Reductase, S-adenosylmethionine synthetase	Muthurajan et al., 2011
*Oryza sativa* L. var. japonica, cv. Prata Ligeiro, IRAT20	Root	2-DE, MALDI-TOF/TOF MS	TI: 463 (Prata Ligeiro) TI: 522 (IRAT20)	GSH-dependent dehydroascorbate reductase 1, Putative superoxide dismutase [Cu-Zn], Triosephosphate isomerase, Ascorbate peroxidase	Rabello et al., 2008
Oryza sativa L. cv. Zhonghua 8, Nipponbare	Leaf sheath	1D-IEF, 2DE-PAGE, CBB stained gels analysed in ImageMaster 2D Elite software	TI: 698	Serine hydroxylmethyltransferase I, Superoxide dismutase, Purative actin depolymerizing factor, Chloroplast ATPase, actin depolymerizing factor	Ali and Komatsu, 2006
**MAIZE**					
*Zea mays* L. (Lc and Io)	Leaves	2-DE, Kepler package	TI: 413 DRP: 78	RAB17 responsive to ABA, enolase, beta-glucosidase, putative cytoplasmic NAD-malate dehydrogenase, phosphoribulokinase, chloroplastic Fru 1,6-bisphosphate aldolase	Riccardi et al., 1998
*Zea mays* L. (Io and F2)	Leaves	IEF, 2-DE scanned, transmittance values into optical density values and relative intensities calculated	DRP: 46	ABA45, oxygen evolving enhancer protein 1 (OEE1), OSR40, malate dehydrogenase, a cystein synthase, phosphoribulokinase	Riccardi et al., 2004
*Zea mays* L. cv. Nongda 108	Embryo	2-DE, MALDI-TOF-TOF/MS	DRP: 111	Malate dehydrogenase, Ascorbate peroxidase, Carbomoylphosphate synthase, Superoxide dismutase, Triosephosphate isomerase	Huang et al., 2012
*Zea mays* L. wild type: Vp5	Leaves	iTRAQ, LC-ESI MS/MS	TI: 7051 DRP: 150	Ribose-phosphate Pyrophosphokinase 4, Photosystem I reaction center subunit XI, Photosystem II reaction center protein L, 4F5 protein family protein, Peroxidase	Zhao et al., 2016a
*Zea mays* L. cv. Zhengdan 958	Leaves	iTRAQ, LC-ESI MS/MS	DRP: 65	RAB17 protein/A3KLI0, Dehydrin/C4J477, ABA-responsiveprotein/K7TFB6, Photosystem I reaction center subunit V/B4G1K9, Asparagine synthetase, Stachyose synthase	Zhao et al., 2016b
*Zea mays* L. wild type: Vp5	Leaves	iTRAQ, LC-ESI MS/MS	DRP: 379 (phosphopeptides)	Phosphoenolpyruvate carboxylase, Na+/H+ antiporter, Ubiquitin ligase protein cop1, arginine serine rich splicing factor rsp41, heterogeneous nuclear ribonucleoprotein r-like	Hu et al., 2015a
*Zea mays* L. cv. Zhengdan 958	Leaves	iTRAQ, LC-MS/MS	DRP: 149 (phosphopeptides)	Serine threonine-protein kinase wnk4-like/K7TZQ1, Phospholipase c/B4FY17, Phosphoenolpyruvate carboxylase/E9NQE1, Probable-trehalose-phosphate synthase/K7V0H0, RAB17protein/A3KLI0, Phosphoenolpyruvate carboxykinase	Hu et al., 2015b
*Zea mays* L. Inbred line B104	Leaves	nanoLC−MS/MS	TI: 536 DRP: 18 (phosphoproteins)	Histidine decarboxylase, adenosyl homocysteinase, Exocyst complex component SEC5A, RANBP2-like and GRIP domain-containing protein	Vu et al., 2016
*Zea mays* L. cv. Tolerant CE704, cv. Sensitive 2023	Leaves	2DE-iTRAQ, LC-MALDI TOF/TOF	DRP: 129 (Tolerant) DRP: 97 (Sensitive)	Dehydrin RAB-17, HSP26, Ribonucleoprotein A, HSP17.4	Benešová et al., 2012
*Zea mays* L. cv. FR697	Xylem sap	2DE, LC-MS/MS	DRP: 39	Xyloglucan endotransg-lycosylase homolog, Peroxidase 52 precursor, Endonuclease, Secretory protein	Alvarez et al., 2008
*Zea mays* L. Inbred tolerant lines 81565 and 200B	Leaves	2DE-IEF-PAGE- MALDI-TOF-MS	TI: 500 DRP: 58	cinnamyl alcohol dehydrogenase, caffeate O-methyltransferase, Cytochrome protein 96A8	Hu et al., 2009
*Zea mays* L. (Io and F2)	Leaves	2DE, LC-MS/MS	TI: 300	methylenetetrahydrofolate reductase, S-adenosyl-L-Met (SAM) synthases, 5-methyltetrahydrafolate	Vincent et al., 2005
*Zea mays* L. cv. Sensitive B73, cv. Tolerant Lo964	Maize Kernels	iTRAQ, LC-MS/MS	DRP: 78	Dehydrin, RAB-17, Late embryogenesis abundant protein LEA 14-A, Stress-inducible membrane pore protein, Protein kinase C inhibitor, Thioredoxin H-type, Superoxide dismutase-4A, ABA-responsive protein,	Yang et al., 2014
**BARLEY**					
*Hordeum vulgare* L. cv. Arta, cv. Keel	Leaves	2-DE, MALDI-TOF/TOF-MS	NA	No significant identification was reported	Rollins et al., 2013
*Hordeum vulgare* L. acc. no. 15141, 15163	Leaves	2-DE, MALDI-TOF-MS	DRP: 22 DRP: 27	Methionine synthase, ATP synthase subunit alpha, glyoxysomal malate dehydrogenase, heat shock protein 90, ATP-dependent Clp protease	Ashoub et al., 2013
*Hordeum vulgare* L. *ssp. Spontaneum* cv. XZ5, cv. XZ54, cv. ZAU3	Leaves	2-DE, MALDI-TOF/TOF-MS	DRP: 38	Ribulosebisphosphate carboxylase small chain clone 512, Glutathione S-transferase 1, ATP synthase beta subunit, Transketolase, chloroplast	Wang et al., 2015
*Hordeum vulgare* L. Jau-83, Frontier-87, Jau-87, Haider-93, Sanober-96 and Soorab-96	Shoots	2-DE, MALDI-TOF-MS, nanospray LTQ XL Orbitrap MS	Sensitive DRP: 31, Tolerant line DRP: 28	ATPase subunit E, photosystem I reaction center II, Ptr ToxA-binding protein, ATP synthase CF1 alpha subunit, glutathione transferase, oxygen evolving complex precursor, HSP70	Kausar et al., 2013
*Hordeum vulgare* L. cv. Amulet	Leaves and crowns	2DE, MALDI-TOF/TOF	DRP: 105	Annexin, Flavoprotein wrbA-like isoform 1, Putative r40c1 protein (ricin B lectin domain), Glutathione peroxidase, Glutathione S-transferase 6, chloroplastic; Methionine synthase, UDP-glucose 6-dehydrogenase	Vítámvás et al., 2015
*Hordeum vulgare* L. cv. Maresi, cv. Cam/B1//CI08887/CI05761	Leaves and roots	2DE, MALDI-TOF and MALDI-TOF/TOF	DRP: 121 leaves, 182 roots	sHSP (17.6 kDa,16.9 kDa) cold-regulated protein (COR),Mitochondrial pyruvate dehydrogenase E1 subunit alpha (PDHA1), NADP dependent malic enzyme (NADP-ME), hydroxy acid oxidase (HAO, also known as glycolate oxidase), oxalate oxidase (OXO), ascorbate peroxidase (APX)	Chmielewska et al., 2016
*Hordeum vulgare* L. cv. Golden Promise, cv. Basrah	Leaves and roots	2DE, MALDI-TOF	DRP: 24 leaves, 45 roots	14-3-3, GTP-binding protein Rab2, cytochrome p450, methionine synthase and S-adenosylmethionine synthase	Wendelboe-Nelson and Morris, 2012
**SORGHUM**					
*Sorghum bicolor* L. Moench cv. 11434, cv.11431	Leaves	2-DE DIGE, MALDI-TOF-MS	DRP: 18 DRP: 23	Methionine synthase, S-adenosylmethionine Synthase, P-(S)- hydroxymandelonitrile lyase, PEPC, PPDK, Hsp60	Jedmowski et al., 2014
**PEARL MILLET**					
*Pennisetum glaucum*. (L.) R. Br. cv. ICTP 8203	Leaves	nanoESI-LC-MS/MS	TI: 1208 DRP: 120	Thioredoxin-dependent peroxidase 2, Glyceraldehyde 3-phosphate dehydrogenase A subunit 2, ribosomal proteins, prohibitin 3, chlorophyll a/b-binding protein	Ghatak et al., 2016
	Roots		TI: 1095 DRP: 25	Cytochrome C1 family, UDP-glucosyl transferase, Annexins, Lipoxygenase, Phosphoinositide-specific phospholipase C (PI-PLC), NAD(P)-linked oxidoreductase superfamily protein	Ghatak et al., 2016
	Seeds		TI: 1299 DRP: 10	LEA, heat shock proteins 70, 21 kDa, threonine synthase	Ghatak et al., 2016

## Wheat

Wheat (*Triticum* spp.) is one of the major food crops in the world and a rich source of glutens and storage proteins in wheat grains (Gill et al., 2004). The genome of the common wheat is 17 Gb and very complex due to numerous polyploidy events that occurred between 8,000 and 10,000 years ago (Gupta et al., 2008; Brenchley et al., 2012). The International Wheat Genome Sequencing Consortium published a chromosome based draft sequence of bread wheat genome (Brenchley et al., 2012) which potentially forms the basis for breeding of biotic and abiotic stress—tolerant varieties.

The proteome map of wheat leaf (Bahrman et al., 2004; Donnelly et al., 2005), flour (Mamone et al., 2000), and amyloplast (Andon et al., 2002) was generated using 2-DE technique. Recently, additional proteomic resources and tools for functional analysis have been developed for wheat (http://www.wheatproteome.org/) (Duncan et al., 2017). A comparative study between young endosperm and mature endosperm revealed a unique set of proteins which is characterized in each developmental stage. A majority of proteins contain 36 thioredoxin targets, most of these targets are also identified in developing endosperm (Hurkman et al., 2004). Vensel et al. identified 250 proteins in the early and late stages of grain development in wheat with a combinatorial approach of 2D-MS (Vensel et al., 2005). Wheat grain specific proteins were identified using 2-DE which was then applied for cultivar identification of flour. These proteins were used as markers to identify wheat cultivars in blended flour composed of more than two or three flours (Skylas et al., 2001; Yahata et al., 2005). A specialized review article related to wheat proteomics provides further information (Komatsu et al., 2014).

Wheat is frequently cultivated in a region which is under water deficit condition. Therefore, it is extremely important to understand drought responsive mechanism of wheat plants. Proteomic analysis in varieties of wheat cultivars has uncovered many drought stress responsive protein candidates. The role of redox regulation under drought stress conditions has been revealed by the proteomic analysis using different tolerant varieties and there susceptible counterparts (Hajheidari et al., 2007). In this study, Khazar—I genotype which is drought tolerant and two drought susceptible genotypes (Afgani and Arvand) were chosen for the comparative analysis using MALDI-TOF/TOF mass spectrometry. This analysis led to the identification of 57 drought responsive proteins out of 121 protein candidates. Among these two third of candidates were thioredoxin targets which included protein candidates like thioredoxin-h, glutathione S-transferase, 1-Cys peroxiredoxin, glyceraldehyde-3-phosphate dehydrogenase and other proteins. Increase in the gliadin storage protein content was also observed in tolerant genotypes compared to susceptible varieties (Hajheidari et al., 2007).

A study performed by Caruso et al. demonstrated the changes in leaf proteome under drought stress condition using 2D-PAGE and MALDI-TOF MS. In this study seven days of water stress were applied to 8 day old plants prior harvesting of leaf and protein extraction. Out of 36 proteins identified, 12 protein spots were up-regulated and 24 protein spots were down-regulated (Caruso et al., 2009). Proteins such as ATP synthase CF1 alpha subunit, phosphoribulokinase were down regulated along with RuBisCo SSU protein under drought stress condition. These results were in agreement with the study performed by Plomion et al. though a study on poplar—which showed that the mRNA encoding this protein is also down-regulated under drought stress condition (Plomion et al., 2006). Further, the identified proteins were also involved in several physiological mechanisms such as ROS scavenging, energy production and stress defense (Plomion et al., 2006).

Thirty three drought-responsive proteins were identified which include ABA-responsive proteins, LEA protein, cyclin-dependent kinase like, zinc finger, transcription factor like MYB, lipid transfer proteins and WRKY domain containing protein (Kamal et al., 2010) in wheat grain of four cultivars (two Chinese cv. China- 108, Yennon-78 and two Japanese cv.Norin-61, Kantou-107). Peng et al. investigated the proteome of roots and leaves under drought and salt stress condition in bread wheat cv. Shanrong no 3 and its parent bread wheat cv. Jinan 177 using MALDI-TOF/TOF-MS. Water stress was applied for 24 h to the plants at two leaf stages by adding 18% (w/v) polyethylene glycol (PEG) 6000 to half strength Hoagland's culture solution. Proteins identified from root and shoot were involved in a variety of functions which include gene transcription, detoxification, signal transduction and carbon and nitrogen metabolism (Peng et al., 2009). In this context, Rizhsky and co-workers demonstrated that gene expression of LSU-RuBisCo was increased strongly under drought stress condition in tobacco. The putative role of this protein in drought tolerance mechanism requires further investigation (Rizhsky et al., 2002).

A proteomic approach using nano LC MS/MS and iTRAQ 8plex in two drought tolerant and one intolerant cultivar from three Australian bread wheat (*Triticum aestivum* L.) cultivars (cv. Kukri, Excalibur, and RAC875) revealed the abundance of CAT and three isoforms of SOD like chloroplastic cytosolic Cu/Zn-SOD and mitochondrial Mn-SOD. These proteins are involved in the survival strategy to avoid excess generation of ROS (Ford et al., 2011). Under drought stress in wheat, adenylate kinase (ADK) level increases after 3 days, but after 6–9 days the ADK level decreases, which is possibly due to cell death (Kamal et al., 2013). Adenylate kinase (ADK) is a small ubiquitous enzyme involved in the metabolism of purine nucleotides and it is essential for cell growth and maintenance.

Quantitative proteomic analysis of roots from two different wheat varieties, Nesser (drought-tolerant) and Opata (drought sensitive) cultivars was performed using a LTQ-Orbitrap Velos mass spectrometer (Thermo Fisher Scientific, Rockford, IL) coupled to an Eksigent nanoLC Ultra (AB Sciex) and iTRAQ 4plex. This study led to the identification of 805 ABA responsive proteins, six LEA proteins and protein phosphatases PP2C in wheat roots under drought stress (Alvarez et al., 2014). Two protein candidates, H+-transporting two sector ATPase and membrane bound ATP synthase subunit b localized in the plasma membrane are dynamically changed under drought stress in chloroplast of wheat (Kamal et al., 2013).

Comparative proteomic analysis in drought tolerant wild wheat variety was studied (*Triticum boeoticum*) by transferring hydroponically grown seedlings at 3-leaf stage into ½ Hoagland solutions with 20% PEG-6000 for 48 h inducing drought stress. Proteomic analysis was performed by 2DE-MALDI-TOF/TOF and revealed 98 protein spots in leaves and 85 protein spots in roots differentially regulated (Drought responsive proteins, DRP's). The DRPs were binned into the function of defense, carbon metabolism, nitrogen, amino acid metabolism, protein metabolism, chaperons, nucleotide metabolism, photosynthesis, and signal transduction. Carbon fixation and photosynthetic ability was decreased in leaves whereas the PPP pathway was enhanced in roots. Glycolysis was down-regulated in root under drought stress (Liu et al., 2015).

Recently, we performed the first large-scale label-free quantitative shotgun proteomics study of pearl millet leaf, root and seed tissue under drought stress (Ghatak et al., 2016). This allows to compare wheat-specific drought stress protein marker with pearl millet specific drought stress marker. One should bear in mind, however, that wheat is a C3 and pearl millet a C4 plant. Therefore, another aim was also to reveal any differences which correspond to these different types of photosynthesis. For comparison we considered the studies performed by Liu et al. (2015), Hajheidari et al. (2007), and Ghatak et al. (2016) (Supporting Table 1). From these studies, it was evident that rubisco subunits, LEA proteins, ATP synthase, ABA responsive proteins and others can be suggested as an protein marker of drought stress conditions for both wheat and pearl millet. These proteins are involved in drought adaptation or tolerance mechanisms which require further investigations. Table 1 enlists all the potential protein candidates in drought stress proteomics studies of wheat (Bazargani et al., 2011; Yang et al., 2011; Ge et al., 2012; Jiang et al., 2012; Kang et al., 2012; Budak et al., 2013; Peremarti et al., 2014; Qin et al., 2014; Wang et al., 2014; Zhang et al., 2014; Cheng et al., 2015, 2016; Giuliani et al., 2015; Hao et al., 2015; Kacem et al., 2016).

## Rice

Rice (*Oryza sativa* L.) is the main staple food for more than half of the world population (http://irri.org/). It is a commonly used cereal crop and model system for molecular biology and genetic research. The completion of its draft genome sequencing (International Rice Genome Sequencing Project, 2005) accelerated proteomics studies in rice (Devos and Gale, 2000; Koller et al., 2002).

There is a significant progress in the proteomic analysis of rice tissues and organelles which include rice embryo (Xu et al., 2012) and endosperm (Komatsu et al., 1993), root (Zhong et al., 1997), etiolated shoot (Komatsu et al., 1999), cell suspension culture (Komatsu et al., 1999), anther (Imin et al., 2001; Kerim et al., 2003), leaf sheath (Shen et al., 2002), and other organs (leaf, stem, and root growth during development; Nozu et al., 2006). A study performed by Tsugita et al., led to the separation of 4892 proteins from nine tissues of rice (leaf, stem, root, germ, dark germinated seedlings, seed, bran, chaff and callus) but only 3% were characterized by short amino acid reads (Tsugita et al., 1994). The first large-scale shotgun proteomics study was reported by Koller et al. (2002) in the cultivar Nipponbare led to the identification of 2528 proteins. This analysis included leaf, root and seed tissue. Various organelles of rice such as golgi bodies (Mikami et al., 2001), mitochondria (Heazlewood et al., 2003), chloroplast and other subcellular compartments were also subjected to proteomic analysis.

Proteomic resources and tools for functional analysis have been developed which includes PhosphoRice: a meta-predictor of rice specific phosphorylation sites (http://bioinformatics.fafu.edu.cn/PhosphoRice; Que et al., 2012), Oryza PG-DB: rice proteome database on shotgun proteogenomics (http://oryzapg.iab.keio.ac.jp/; Helmy et al., 2011) and PRIN: a predicated rice interactome network (http://bis.zju.edu.cn/prin/; Gu et al., 2011). These bioinformatic resources are very helpful for proteomic analysis in order to categorize the identified proteins into a functional category along with their dynamic interaction network in rice and other plants.

Proteomic analysis of rice tissues under drought stress includes seedling (Shu et al., 2011), anther (Liu and Bennett, 2011), peduncles (Muthurajan et al., 2011), extracellular matrix (Pandey et al., 2010), rice leaves at different stages and genotypes (Xiong et al., 2010; Ji et al., 2012; Mirzaei et al., 2012a) and root (Mirzaei et al., 2012b). Recently, comparative proteomic studies were performed on *Oryza sativa* L. cv. IR64 considering well-watered, drought and partially dried root samples (Mirzaei et al., 2012b). Quantitative label free shotgun proteomic analysis resulted in the identification of 1487 non-redundant proteins. Similarly, a study performed on 35 days old seedlings of *Oryza sativa* cv. Nipponbare (drought sensitive cultivar) after moderate drought stress and extreme drought stress, followed by re-watering for 3 and 6 days lead to the identification of 1548 non-redundant protein using label free shotgun proteomics (nano-LC-MS/MS). It was also observed that regulation of aquaporins, small GTPases, and V-ATPases under drought stress and recovery phase are closely involved in drought signaling and drought responses (Mirzaei et al., 2012a). Several specialized review articles related to rice proteomics have been published and are recommended for further reading (Rakwal and Agrawal, 2003; Agrawal and Rakwal, 2006; Komatsu, 2006; Komatsu and Yano, 2006; He and Yang, 2013; Singh and Jwa, 2013; Kim et al., 2014).

The apoplastic proteome was investigated under drought stress in 4 weeks old rice (*Oryza sativa* L. var. Rasi) seedlings using 2-DE (Pandey et al., 2010). In total 192 proteins showed changes in abundance involved in functions such as carbohydrate metabolism (cytosolic 6 phosphogluconate dehydrogenase, putative ribose 5-phosphate isomerase, sedoheptulose 1,7- bisphophatase, enolase, etc.), cell defense and rescue (Cu/Zn SOD, L-APX, GSH-dependent dehydroascorbate reductase, thioredoxin, POD), cell wall modification, cell signaling (GDP dissociation inhibitor, GF1-c protein, nucleoside-diphosphate kinase) and molecular chaperons (DnaK type molecular chaperone, putative peptidyl prolycis trans isomerase). The plant cell apoplast is a dynamic compartment involved in many processes which include cell growth, development, signaling and biotic/abiotic stress. There is a strong regulation of apoplastic proteins in response to salt, temperature, wounding and pathogen invasion (Guo and Song, 2009).

Salekdeh et al. measured the proteome of rice leaves under drought stress in two genotypes, lowland *Indica* (IR62266-42-6-2) and up-land *Japonica* (CT9993-5-10-1-M) using 2-DE. The study also included the identification of drought responsive protein candidates after recovery. Under stress conditions, 42 protein spots showed changes in abundance, among these 27 proteins exhibited different response pattern in two genotypes. Sixteen drought responsive proteins were identified using MALDI-MS or ESI-MS/MS which include increased levels of S-like RNase homolog, actin depolymerizing factor and RuBisCo activase, whereas isoflavone reductase protein was decreased (Salekdeh et al., 2002). Protein candidates like Cu-Zn superoxide dismutase showed increased levels in both the genotypes. The gene encoding protein Cu-Zn SOD is induced under drought stress in roots and leaves (Kaminaka et al., 1999; Plomion et al., 2006).

Proteomic and phosphoproteomic analysis was performed on 2 weeks old rice (*Oryza sativa* L. cv. Nipponbare) plants under drought stress. Late embryogenesis abundant (LEA) proteins and chloroplast precursor Cu-Zn SOD were up-regulated and a Rieske Fe-S precursor protein was down-regulated under stress condition (Ke et al., 2009). Down-regulation of Rieske Fe-S precursor protein under drought stress was also demonstrated by Salekdeh et al. (2002). Researchers also identified 10 phosphoproteins under drought stress which include: NAD-malate dehydrogenase, OSJNBa0084K20.14 protein, abscisic acid and stress-inducible protein, ribosomal protein, drought-induced S-like ribonuclease, ethylene-inducible protein, guanine nucleotide-binding protein beta subunit-like protein, r40c1 protein, OSJNBb0039L24.13 protein and germin-like protein 1. Seven of these phosphoproteins have not previously been reported to be involved in rice drought stress (Ke et al., 2009). Protein candidates like putative r40c1 and germin like protein 1 were less phosphorylated under drought stress. The identification of drought responsive phosphoproteins provides valuable insights to understand regulatory mechanism of stress responses in crop plants because phosphorylation is one of the most important post translational modifications (PTMs) involved in early stress responses and the regulation of almost all cellular adaptation processes (Glinski and Weckwerth, 2006; Wolschin et al., 2006). Drought stress has detrimental effect on plant reproductive stages. Liu and Bennett performed rice anthers proteomics under drought stress. They considered two genotypes, Morobereken (drought tolerant) and IR64 (drought sensitive), and reported 35 proteins of which eight were drought induced which includes glyceraldehyde-3-phosphate dehydrogenase, β-expansin and actin binding protein (Liu and Bennett, 2011). Table 1 provides details of the discussed proteomic studies performed in rice under drought stress (Ali and Komatsu, 2006; Zang and Komatsu, 2007; Rabello et al., 2008, 2014; Shu et al., 2011; Dong et al., 2014; Mirzaei et al., 2014; Agrawal et al., 2016; Wu et al., 2016).

## Maize

Maize (*Zea mays* L.) belongs to the most cultivated cereal crops worldwide together with wheat and rice. An estimated production for the year 2012 is 839 million tons according to United States Department of Agriculture (USDA). Maize is the staple food for substantial part of world's population as well as a major source of animal feed (Strable and Scanlon, 2009).

Maize is a cross pollinated species which possess an extraordinary level of genotypic diversity helpful to investigate evolutionary processes and to provide natural genetic variation for brewing (Doebley, 2004), recombination and transposition (McClintock, 1930), heterosis, genomic imprinting (Kermicle, 1970) and epigenetic phenomena (Brink, 1958).

The maize genome sequence of the B73 line (http://www.maizesequence.org) has opened wide areas of research (Schnable et al., 2009). All the genetic information is accessible via public domain repositories like MAizeGDB (http://maizegdb.org/), PlantGDB (http://www.plantgdb.org/ZmGDB), and Plant Proteome Database (http://ppdb.tc.cornell.edu), TIGR Maize Database (http://maize.jcvi.org), Maize Assembled Genomic Island (http://magi.plantgenomics.iastate.edu/). Earlier, maize proteome projects used peptide mass fingerprinting for identification of proteins which rely on ESTs databases. The proteome search became more feasible after genome sequencing (Bennetzen and Freeling, 1997; Bennetzen and Ma, 2003). Maize tissues analyzed by proteomics comprise leaf tissue (Porubleva et al., 2001; Majeran et al., 2010), endosperm (Méchin et al., 2004), egg cell, root hair (Nestler et al., 2011), primary root (Hochholdinger et al., 2005), primary root pericycle (Dembinsky et al., 2007), root tip (Chang et al., 2000), starch granules (Pechanova et al., 2010; Koziol et al., 2012) and seed flour (Albo et al., 2007).

Proteome analysis of maize egg cells and the zygote using 2-DE/MS indicated that maize egg cell is rich in enzymes of energy metabolism. Annexin was also identified from egg cell and zygote. It is involved in exocytosis of cell wall material. Since the process of fertilization increases the levels of cytosolic calcium, the role of particular proteins in calcium metabolism could be defined. Zhu et al. analyzed the cell wall proteome in the maize primary root elongation zone. The protein candidates such as endo-1,3;1,4-beta-D-glucanase and alpha-L-arabinofuranosidase were identified which act on major polysaccharides in root development. The plant chloroplast is an important organelle harboring the photosynthetic apparatus and genes encoded either by a chloroplast plastid or the nuclear genome (Zhu et al., 2006). Therefore, organelle proteomics is a decisive technology also to reveal targeting of proteins to chloroplasts (Glinski and Weckwerth, 2006). Lonosky and co-workers identified 26 unique proteins from the maize chloroplast during five stages of leaf greening (0–48 h post illumination; Lonosky et al., 2004). Ma et al. reported a comprehensive proteome analysis of root mucilage, an exudate that is continuously secreted by maize primary root tips and serves as a significant source of carbon for rhizospheric microbes. Mucilage from 3 day old primary roots was subjected to proteomic analysis using 1DE and nanoLC-MS/MS approach and led to the identification of 2848 proteins. Most of the identified proteins were involved in the function of energy metabolism, amino acid and lipid metabolism. Additionally, proteins involved in secondary metabolism of terpenes, flavonoids and phenolic were found to be abundant. However, 16% of proteins were involved in a secretory pathway (Ma et al., 2010). Details of maize proteomic studies from the time period 1987–2012 have been reviewed extensively (Pechanova et al., 2013).

Several proteomic studies have been carried out in maize that determines drought stress responses (Benešová et al., 2012; Yang et al., 2014; Hu et al., 2015a,b; Vu et al., 2016; Zhao et al., 2016a,b). Riccardi et al. studied the leaf proteome in two different genotypes of maize (referred as LC and Io). Water stress was applied for 10 days until the plants were aged to five leaf stages. For proteomic analysis the sixth leaf was considered and 413 protein spots were quantified of which 78 were affected under drought stress. Nineteen differentially expressed proteins were identified under drought stress. The identified proteins which were up-regulated under drought stress include RAB 17, phosphoribulokinase and caffeate *O*-methyltransferase, COMT, glutamate semialdehyde aminotransferase (GSAAT), β-glucosidase, chloroplastic fructose bisphosphate aldolase, and ferritin were also up-regulated under stress conditions. Most of the identified proteins were binned into the function of photosynthesis and lignin biosynthesis pathway (Riccardi et al., 1998). In another study, changes in the leaf proteome of maize (in two different genotype *Io* and *F2*) under water deficit conditions were measured. Protein candidates comprised oxygen evolving enhancer (OEE) protein 1, malate dehydrogenase and ABA stress ripening (ASR) proteins. The identified proteins were observed to have different level of induction in different genotypes under stress condition. Therefore, based on this study, authors concluded that in order to define a protein marker it is extremely important to consider effects of genetic variation which have direct effect on the accumulation of stress induced proteins (Riccardi et al., 2004).

Xylem sap of maize (*Zea mays* cv. FR697) was subjected to proteomic (using LC–MS/MS) and metabolomic analysis under drought stress condition; ABA and 6-benzylaminopurine were significantly up-regulated in stressed plants (Alvarez et al., 2008). Other compounds such as glutamate/glutamine, serine and threonine also showed increased abundance under stress condition. However, there was regulation decrease of *trans zeatin* and its conjugated form (*trans zeatin* riboside) under drought stress condition. This study also suggested signaling mechanisms by which root to shoot communication changes under water deficit condition. Further 39 proteins were identified which differentially expressed under stress condition and binned in the function of cell wall metabolism and defense mechanism (which includes: peroxidase, xyloglucan endotransglycosylase, polygalacturonase inhibitor and pectin methylesterase and plant defense mechanisms such as thaumatin-like pathogenesis-related protein, zeatin-like protein, cupin family protein, putative germin A, class IV chitinase and β-1,3-glucanase).

The effect of drought stress in leaf elongation was studied in maize using a proteomic strategy by Vincent et al. (2005). This study demonstrated the accumulation of two isoforms (acidic protein COMT 1 and less acidic protein COMT 2) of caffeic acid/5-hydroxyferulic 3-Omethyltransferase (COMT) in growing leaf in well irrigated plants (at 10–20 cm). In contrast, under drought stress no accumulation was observed. It was also reported that lignin content in leaf under stress condition is lowered compared to control plants. These findings were supported by a study which also reported two key enzyme of lignin biosynthesis differentially expressed in maize leafs under drought stress (Hu et al., 2009). Lignin content in plants is mainly associated with the function of mechanical support and defense against biotic and abiotic stress (Boudet, 2000). These results are extremely interesting to provide valuable information that lignin content of leaf is a useful parameter to evaluate drought tolerance in maize and it can be a potential molecular selection marker for drought tolerance (Boudet, 2000).

Desiccation tolerance in maize (*Zea mays* L. cv. Nongda 108) embryos during their development and germination were investigated using proteomic analysis (2-DE, MALDI-TOF and TOF/TOF MS). In this study eleven proteins involved in drought tolerance were identified which include late embryogenesis abundant (LEA) protein, EMB 564, globulin 2, putative cystatin, class I HSP, NBS-LRR resistance like protein RGC456 etc. These proteins are stored during embryo maturation but their abundance decreases during embryo germination (Huang et al., 2012).

All discussed studies and identified protein marker are listed in Table 1.

## Barley

Barley (*Hordeum vulgare* L.) is an important crop in dry and marginal climatic environment. Barley has a wide tolerance capacity for drought and other abiotic stresses. Hence barley is widely used for breeding in the specific environmental condition or for the specific adaptation to abiotic stress in geographically distinct areas of the globe. For example, barely germplasm for marginal environment from west Asia and northern Africa by International Center for Agriculture Research in Dry Areas, Syria (ICARDA) has shown good adaptation in the southern Australian environments and vice versa (Shakhatreh et al., 2001). This adaptation of genetically diverse germplasm to extreme climatic condition in a wide geographical range can be exploited for germplasm exchange and breeding as well as providing interesting insights to the genetic basis of stress adaptation.

Stress adaptation of barley is mainly attributed to its plasticity of morphological traits (for e.g., biomass production, plant growth, tiller number and peduncle extrusion, Shakhatreh et al., 2010). Physiological parameters such as relative water content and chlorophyll fluorescence parameter under stress condition in barley are also characterized by genetic variations (Oukarroum et al., 2007; Ahmed et al., 2013). Wide ranges of transcriptome information are available on drought stress as well as in combination of other stresses (Talamè et al., 2007; Guo et al., 2009). As the level of mRNA does not well correlate with protein levels, identification of proteomes complement missing information in DNA or mRNA analysis. In 2012 the barley genome was sequenced (The International Barely Sequencing Consortium) and became an important model system for proteomics, gene function analysis and stress response. The universal nuclear protein database of barley provides information of proteomic datasets obtained from barley nuclei (http://barley.gambrinus.ueb.cas.cz/; Blavet et al., 2017). A few specialized articles details review proteomic analysis in barely (Finnie and Svensson, 2009; Finnie et al., 2011).

Rollins et al. measured the leaf proteome under heat and drought stress using two genotypes (cv. Arta and cv. Keel). Both the genotypes were genetically diverse but adapted to same drought prone environments. This study provided the information on molecular and phenotypic changes under drought and heat stress. Overall, drought stress showed a stronger effect on morphological traits (for e.g., variation in grain yield, variation in biomass, variation in no. of spikes, variation in relative water content, and variation in photosynthetic performance index) compared to physiological traits. Under drought stress the leaf proteome did not reveal significant changes in protein abundance compared to the control. These results were in contrast to the transcript level where large numbers of transcripts were differentially expressed under drought stress. Hence it can be concluded that barley has adapted to non-lethal drought using avoidance mechanisms which include reduction in the growth of plants in order to maintain cellular homeostasis (Rollins et al., 2013).

Comparative proteomic analysis of two cultivars (#15141, drought tolerant and #15163, drought sensitive) led to the identification of differentially expressed proteins under drought stress (Ashoub et al., 2013). Based on MALDI TOF-MS analysis the identified proteins were categorized into functional groups. Proteins involved in the group of metabolism include lipooxygenase, NADP-dependent malic enzyme, sucrose synthase and betain aldehyde dehydrogenase which were up-regulated only in #15163 cultivar. The levels of methionie synthase were up regulated in both the cultivars. Considering energy metabolism, several proteins showed alteration in there abundances under drought stress, e.g., chloroplastic ATP synthase subunit alpha showed increased abundance in both cultivars wereas chloroplastic transketolase was down-regulated in both cultivars. Other protein candidates like HSP 90, ATP dependent Clp protease and protein disulfide isomerase were up-regulated in both cultivars under drought stress. Enzymes related to detoxification of cells and controlling photorespiration were increased under drought tolerant cultivar while there was no alteration seen in the drought sensitive cultivar. Proteins associated with the production of osmotically active compounds were increased in the sensitive genotype indicating that drought stress triggers stronger osmotic responses in the sensitive genotype (Ashoub et al., 2013). Similarly comparative proteomic analysis of Tibetan wild barley genotype (drought tolerant XZ5 and drought sensitive XZ54) and cv. ZAU 3 was performed (Wang et al., 2015). This study led to the identification of 38 drought tolerance associated proteins which were binned into the functional category of photosynthesis, stress response, metabolic process, energy and amino acid biosynthesis. Protein candidates like melanoma- associated antigen p97, type I chlorophyll a/b binding protein b, ATP synthase CF1 beta subunit, ribulosebisphosphate carboxylase large chain were expressed or up-regulated exclusively only in XZ5 compared to XZ54. Out of 38 proteins 20 proteins were up-regulated in XZ5 and simultaneously down-regulated in XZ54, which highlights the significance of “drought tolerance associated specific proteins” in drought tolerance in Tibetan wild barely (Wang et al., 2015).

Proteomic analysis identified 24 differentially expressed proteins in leaf and 45 in root using MALDI-TOF MS considering two barely varieties (UK, golden Promise and Iraq, Basrah). When compared to control plants (unstressed plants) 66 proteins were differentially expressed in leaves and 77 in roots (DIGE analysis) (Wendelboe-Nelson and Morris, 2012). Further studies in barley under drought stress are mentioned in Table 1 (Kausar et al., 2013; Vítámvás et al., 2015; Chmielewska et al., 2016).

## Sorghum

Sorghum (*Sorghum bicolor*) is a multipurpose crop belonging to the Poaceae family; it is a C4 plant with high photosynthetic efficiency and productivity (Alkaraki et al., 1995). Sorghum is one of the five major cultivated cereal crops in the world. It has high economic importance and is used as food (grain), feed (grain and biomass), fuel (ethanol production), fiber (paper), fermentation (methane production), and fertilizer (utilization of organic by-products). Sorghum is a staple food that supplies a major proportion of calories and proteins to large segments of populations in the semi-arid tropical regions of Africa and Asia. Sorghum bran and spent grain as by-products are rich in protein and are generally sold at a cheap price for animal feed and also used as biopolymer for food packaging (Cuq et al., 1998). Sorghum grain has a protein content varying from 6 to 16%, with an average of 13% (Lasztity, 1996). Sorghum grain proteins can be broadly classified into prolamin and non-prolamin proteins. Kafirins, the major storage proteins, are classified as prolamins, and as such, they contain high levels of proline and glutamine and are soluble in non-polar solvents such as aqueous alcohols (Shewry and Tatham, 1990). Kafirins account for 77–82% of the protein in the endosperm, whereas non-prolamin proteins (namely, albumins, globulins, and glutelins) make up about 30% of the proteins (Belton et al., 2006). Sorghum proteins have been also used to produce biodegradable films (Buffo et al., 1997). In 2009, the sorghum genome was published (www.plantgdb.org/SbGDB/; Paterson et al., 2009), which has facilitated omics analysis. Recently, a sorghum whole genome co-expression network database was made available (http://structuralbiology.cau.edu.cn/sorghum/index.html) which can propose gene functions by gene associations (Tian et al., 2016).

A study performed by Jedmowski et al. on drought stress analyzed 5 leaves stages in two genotypes of sorghum (Accession number #11434, drought tolerant, and accession number #11431, drought sensitive) using 2D-DIGE. Differentially expressed proteins were analyzed by Matrix assisted laser desorption ionization time of-flight mass spectrometry. DEP's were involved in energy metabolism. Furthermore, chaperons were among the most prominent features to elucidate the differences between the drought tolerant and sensitive accessions. Other metabolism related proteins included methionine synthase, S-adenosylmethionine synthase, and P-(S)-hydroxymandelonitrile lyase. Methionine synthase is upregulated in both genotypes (#11434 and #11431) under drought stress, following recovery, expression levels remained upregulated in #11434 and returned to control values in #11431. Pyruvate phosphate dikinase (PPDK) was upregulated under drought stress and recovery in #11434. However, the MALDI-TOF-MS data analysis did not indicate if it is the cytoplasmic or chloroplastic form of the enzyme (Jedmowski et al., 2014).

## Pearl millet

Pearl millet (*Pennisetum glaucum*) is one of the most resilient cereal crops with a very large genome size (~2,400 Mb) and short life cycle (Vadez et al., 2012). It is a highly cross-pollinated diploid (2n = 2x = 14) with excellent photosynthetic efficiency (C4 photosynthesis like maize and sorghum) and biomass production potential. Our knowledge about pearl millet C4 photosynthesis is limited, especially on the encoding genes and their protein levels. Pearl millet has valuable stock of genetic and genomic resources which can be explored using various DNA-based molecular markers including RFLP (Liu et al., 1994); sequence-tagged sites (STS) (Devos and Al, 1995); AFLP (Allouis et al., 2001); SSRs (Qi et al., 2004); diversity arrays technology (DArTs) (Supriya et al., 2011); SNPs and conserved intron spanning primer (CISP) markers (Sehgal et al., 2012); mapping populations, and DNA-marker based linkage maps (Morgan et al., 1998). Recently an international organization ICRISAT have initiated sequencing of pearl millet genome in collaboration with various international partners (IPMGSC consortia).

The developed genetic map is not only useful for detection but also for breeding of promising QTLs for various traits (Jones et al., 2002; Bidinger and Blummel, 2007; Bidinger et al., 2007; Blummel et al., 2007) which includes terminal drought tolerance (Yadav et al., 2002, 2004; Kholova et al., 2012), components of drought adaptation (Kholova et al., 2012), grain and stover yield (Yadav et al., 2003). This information is valuable to understand complex relationships between pearl millet and other cereal crops (Devos and Gale, 2000).

A major QTL for terminal drought tolerance (DT) in pearl millet has been identified and mapped on linkage group 2 (LG 2) using segregating populations derived from two independent crosses between H 77/833-2 and PRLT 2/89-33, and ICMB 841 and 863B (Yadav et al., 2011). This QTL on LG 2 has been considered a major target for marker aided selection (MAS) for improving grain yield and grain stability across variable terminal stress conditions in pearl millet (Yadav et al., 2011). This DT-QTL has also been found to confer high levels of abscisic acid (ABA) in leaf and limiting transpiration rates in drought tolerant pearl millet lines (Kholovà et al., 2010). Performance of LG 2 major QTL for terminal drought tolerance was also assessed under salt stress and interestingly it was found to have enhanced growth and productivity traits under saline and alkaline conditions by limiting Na^+^ accumulation in pearl millet leaves (Sharma et al., 2011). Another study established that the drought tolerant parent (PRLT 2/89-33) and two QTL-near isogenic lines (NILs) recorded higher yield under salinity stress at post-flowering growth stages as compared to drought sensitive parent (Sharma et al., 2014).

Identification and characterization of stress responsive genes and their proteins from pearl millet will not only help in understanding stress regulated pathways but will also help in designing strategies for improving stress tolerance/resistance of pearl millet as well as other related crop plants. There has been only one report on transcriptome analysis of pearl millet under abiotic stress conditions (Mishra et al., 2007). A total of 2,494 differentially regulated transcripts in response to drought, salinity and cold stress were identified and the study indicated the existence of a complex gene regulatory network that differentially modulates gene expression under various stresses (Mishra et al., 2007).

The use of particle bombardment for transformation on pearl millet using plasmid pMON8678 was reported (Taylor and Vasil, 1991). Later several research groups reported the use of biolistic transformation on pearl millet with different target tissues and transgenic to develop a variety resistant fungal pathogen (Ceasar and Ignacimuthu, 2009). However, till date there has been no report on genetically engineered abiotic stress tolerant pearl millet variety. Recently, agrobacterium mediated transformation using shoot apices has been reported in pearl millet (Jha et al., 2011).

A study performed by Choudhary et al. identified and validated differentially expressed genes in response to drought stress in *P. glaucum* by Suppression Subtractive Hybridization (SSH) analysis. Twenty-two days old seedlings of *P. glaucum* cultivar PPMI741 were subjected to drought stress by treatment with 30% Polyethylene glycol (PEG) for different time periods. Total RNA was isolated to construct a drought responsive subtractive cDNA library through SSH, 745 ESTs were assembled into a collection of 299 unigenes having 52 contigs and 247 singletons. All 745 ESTs are submitted to ENA-EMBL databases (Accession no: HG516611-HG517355). After analysis, 10 differentially expressed genes were validated by qRT-PCR namely abscisic stress ripening protein, ascorbate peroxidase, inosine-5′-monophosphate dehydrogenase, putative beta-1, 3-glucanase, glyoxalase, Rab7, aspartic proteinase oryzasin, DnaJ-like protein, and calmodulin-like protein. The identified ESTs have revealed a major portion of stress responsive genes that may provide a basis to investigate the tolerance of pearl millet to drought stress (Choudhary et al., 2015). In Supporting Table 2 we provide a comparison between these EST markers and our drought stress proteomic markers identified in a recent large-scale proteomics study (Ghatak et al., 2016). A shotgun proteomics approach (GEL-LC-Orbitrap-MS) was employed by us to investigate putative protein markers from different tissues (root, seed, and leaf) under drought stress condition (Ghatak et al., 2016). In total 2281 proteins were identified and quantified from the harvested root, seed and leaf of which 120 proteins in leaves, 25 proteins in roots and 10 proteins in seeds showed significant changes under drought stress (drought responsive proteins; DRP's). These DRP's were also searched for nearest orthologs in rice and sorghum for functional categorization. These proteins were categorized into heat shock proteins (HSPs), molecular chaperones, storage proteins and late embryogenesis abundant (LEA) with increased levels in seeds. During drought stress, signaling pathways play a very important role for the plant adaptation to stress in order to maintain cellular homeostasis. In our study we found signaling proteins such as GTP binding protein, leucine rich transmembrane protein kinase, calreticulin, calnexin, 14-3-3 protein, phosphoinositide's specific phospholipase C (PI-PLC) showing increased levels under stress conditions. Proteins related to ABA and other hormone metabolism (Auxin, Jasmonate) were observed to be decreased in root and leaf under water deficit condition compared to control. We have no clear explanation, however, observed a higher activity of deep penetrating roots of pearl millet under drought stress. This might negatively affect hormone metabolism and thereby impairing the root to shoot communication (Skylas et al., 2001). However, further studies are underway to investigate this phenomenon in more detail.

One of the C_4_ enzymes, phosphoenolpyruvate carboxylase (PEPC) has been widely studied in many plants and notably in two grass species, sorghum and maize (Ku et al., 1996; Sheen, 1999). A small multigenic family encodes the PEPC enzymes, which are involved in various functions (Lepiniec et al., 1994; Gehrig et al., 2001). These enzymes fix the atmospheric CO_2_ in C_4_ photosynthetic pathway. Each PEPC isoform is specifically expressed (or constitutive housekeeping) and accumalated (i.e., gene transcript) in different tissues (for example green leaf, roots) (Lepiniec et al., 1994; Gehrig et al., 2001). PEPC enzymes are also studied to understand the origins of C_4_ and CAM photosynthesis (Gehrig et al., 2001; Besnard et al., 2002). In grasses, like wheat (González et al., 1998, 2002), sugarcane (Albert et al., 1992), maize (Izui et al., 1986), and sorghum (Crétin et al., 1991; Lepiniec et al., 1991) the isoforms of PEPC are well characterized. Several PEPC isoforms were also identified in our recent proteomic analysis of pearl millet and showed differential concentration levels in leaf, root and seed tissues under control and drought stress condition (see Supporting Figure 1; Ghatak et al., 2016). Supporting Table 1 provides accessions of PEPC isoforms in pearl millet and their corresponding orthologs in arabidopsis. These data provide a framework to investigate C4 photosynthesis in pearl millet in more detail.

## Summary and simplified scheme of drought stress proteome and signaling perception and transduction responses in cereal crops

Plant responses to stress are dynamic processes able to enhance tolerance/resistance mechanisms and to establish metabolic homoeostasis under extreme environmental conditions. At proteome level, profound alterations are observed in the protein abundances between control and stressed plants as well as between different genotypes (Table 1). These changes cannot be revealed by classical RNAseq or any other genomic technology and are therefore highly complementary to existing genome-wide RNAseq or EST data. We have summarized drought responsive proteins (DRP's) in Table 1 thereby providing an reference point to develop regulatory models for drought stress responses in cereal crop plants, such as wheat, rice, maize, barley, sorghum, and pearl millet. Some of specific protein changes were identified in all the cereal crops discussed in this review and so we summarized selected responses schematically in Figure 4.

Regulatory proteins play an important role in regulating the alteration under water deficit conditions which represents some of the most important targets for crop improvement e.g., transcription factors (like WRKY, MYB), kinases, 14-3-3 and G-proteins which can modulate whole signaling pathway and therefore having profound effects on plant growth and development. These factors were up-regulated in the studies reported. Protein phosphorylation plays an important role for signal perception and transduction under drought stress. Several kinases and phosphatases such as CDPK, SnrK2, PP2C, and other signaling proteins like calreticulin are regulated leading to stress adaptation and maybe tolerance mechanisms like stomata closure. Regulation of ABA under drought is well discussed in many studies, however, the dependency on C3 and C4 photosynthesis needs to be further addressed (Kim et al., 2010; Figure 4).

Stress acclimatization is an energy consuming process which is indicated by alterations in energy metabolism. Therefore, increased abundance of the enzymes involved in glycolysis is observed and reported for e.g., GADPH, TPI, mitochondrial NAD^+^-dependent malate dehydrogenase (MDH) (Krebs's cycle) and components of mitochondrial ATP synthase. Several photosynthetic proteins showed up-regulation under drought stress like rubisco LSU, LHC I, and LHC II proteins, proteins of the oxygen-evolving complex (OEC), carbonic anhydrase and dehydrins determining their protective functions.

Drought stress leads to enhanced risk of protein damage due to cellular instability/imbalances. Therefore, several proteins with protective mechanisms show increased levels, e.g., enhanced accumulation of HSPs, namely HSP 70, 90, and some sHSPs (Chaturvedi et al., 2015). Disturbed cellular metabolism leads to the oxidative damage and hence increase in ROS scavenging enzymes was also observed (for e.g., SOD, GST, 2-cyc Prx, Trx-h, CAT, and APX) which is one of the common and practical features in the plants under drought stress.

Drought stress also affects cellular transport and membrane properties; hence, there is enhanced need of ion transporters, which can take proper signals for appropriate functioning. Increased abundance of V-ATPase was reported in the studies. Aquaporins such as H^+^ATPase or monosaccharide sensing protein 2, PIPs were also reported in response to drought stress. The enzyme involved in lignin metabolism such as caffeoly-coenzyme A O-methyltransferase (COMT) was also reported indicating its potential role in cell wall remodeling under drought stress.

## Conclusion and future prospects

Cereals are the major food crops which are necessary for the survival of the entire human population across the globe. Rice, wheat, maize, barley, sorghum, and pearl millet are major cereal crops contributing ~92% of total crop production across the agricultural field and in the glass house in almost all countries (http://www.icrisat.org/tag/icrisat-annual-report-2015/). Not only humans but also livestock farming depend on these crops. Considering all the drastic global climatic changes, our food security and its distribution is a big question. Marker Assisted Breeding (MAB) and SMART breeding techniques are key in agricultural productivity.

Proteomics is one such tool to improve SMART breeding. Comparative proteomics analysis in different plant species lead us to the information related to various drought related mechanisms and processes which cannot revealed by transcriptomics or other genomic technologies. Application of proteomics to identify and validate drought and heat responsive proteins holds a key feature in marker assisted breeding techniques in future.

The studies on plant proteomes in relation to abiotic stress are rather limited. Nevertheless, there is a increasing number of studies in the last 3 years due to extreme heat and drought conditions affecting plant growth and productivity and necessitating our better understanding of underlying biochemical, physiological and morphological principles. Drought is a major area of concern for agriculture production, food availability and quality. It disturbs the universal food chain resulting in weakness and death of humans and animals. As a plant researcher one primary aim should be to translate basic research programs and results into breeding programs together with breeders for more tolerant varieties of plants which can combat drastic drought conditions across the globe.

In order to support comparative proteomic approaches a large number of protein reference maps for various tissues are already available for model cereal crops. We need to urgently generate proteome maps from cereal crops that will greatly help in understanding various cellular processes and signaling pathways. Moreover, it is now possible to uncover the regulation of these processes by studying the post-translational modifications (PTM). Quantitative proteomics approaches such as phosphoproteomics and other OMICS analysis such as metabolomics, transcriptomics, lipidomics will further improve the understanding of molecular mechanisms associated with water stress responses (Weckwerth, 2011a). The integration of genomics, proteomics, transcriptomics, and metabolomics is important for future research in green systems biology to improve its application in cereal crops (Weckwerth, 2011a). In this review, the available proteomics data sets for cereal crops under drought stress have been summarized providing a basic starting point for a comparative overview of the drought stress proteome variations in cereal crops.

## Author contributions

AG, PC, and WW conceived and wrote the manuscript.

### Conflict of interest statement

The authors declare that the research was conducted in the absence of any commercial or financial relationships that could be construed as a potential conflict of interest.

## Supplementary material

Supporting Figure 1 **Phosphoenolpyruvate carboxylases (PEPCs) isoforms in Pearl Millet (Ghatak et al., 2016)**.

Supporting Table 1 **Comparison between drought stress EST markers (Choudhary et al., 2015) and drought stress proteomic markers (Ghatak et al., 2016)**.

Supporting Table 2 **Comparative proteomic analysis between wheat and pearl millet**.
