# Supplementary figures and images for: Cereal Crop Proteomics: Systemic Analysis of Crop Drought Stress Responses Towards Marker-Assisted Selection Breeding
Source: Front Plant Sci. 2017 Jun 2;8:757. doi: 10.3389/fpls.2017.00757 (PMC5454074; doi:10.3389/fpls.2017.00757)

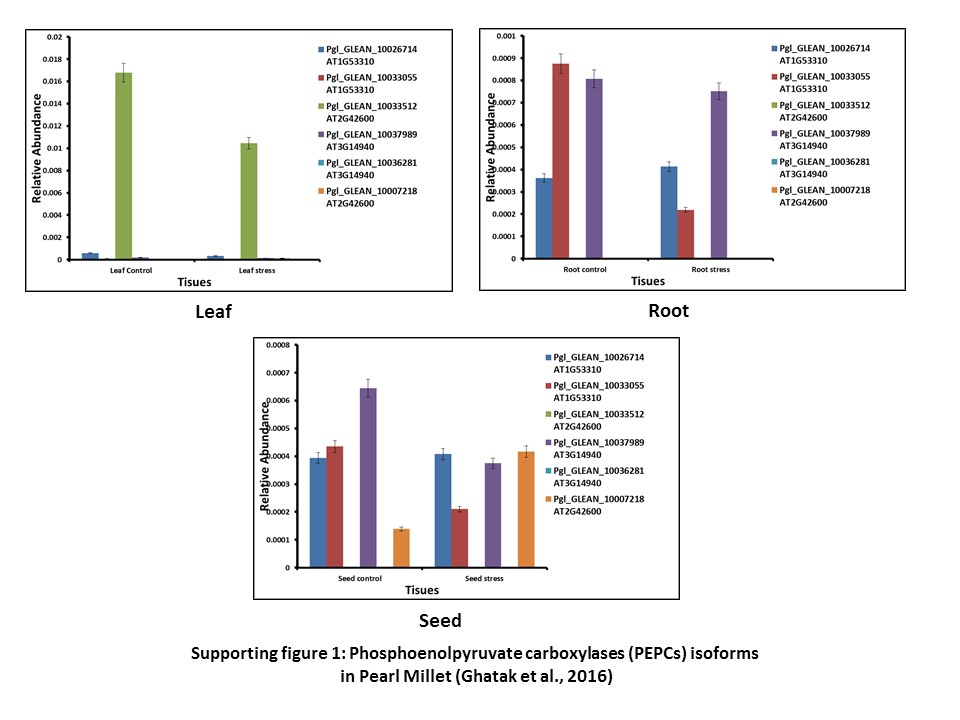

Supplement: Supporting Figure 1 — Phosphoenolpyruvate carboxylases (PEPCs) isoforms in Pearl Millet (Ghatak et al., 2016). [file Image1.JPEG]
